# Removal of Methylene Blue Dye from Aqueous Solutions Using Polymer Inclusion Membrane Containing Calix[4]pyrrole

**DOI:** 10.3390/membranes14040092

**Published:** 2024-04-17

**Authors:** Anna Nowik-Zajac, Iwona Zawierucha, Jakub Lagiewka, Karolina Jaksender, Katarzyna Witt, Grzegorz Malina, Vira Sabadash

**Affiliations:** 1 Institute of Chemistry, Jan Dlugosz University in Czestochowa, Armii Krajowej 13/15, PL 42200 Czestochowa, Poland; i.zawierucha@ujd.edu.pl (I.Z.); jakub.lagiewka@doktorant.ujd.edu.pl (J.L.); karolina.jaksender74@gmail.com (K.J.); 2 Faculty of Chemical Technology and Engineering, Bydgoszcz University of Science and Technology, Seminaryjna 3, PL 85326 Bydgoszcz, Poland; wittkatarzyna@pbs.edu.pl; 3 Department of Hydrogeology and Engineering Geology, AGH University of Krakow, Mickiewicza 30, PL 30059 Cracow, Poland; gmalina@agh.edu.pl; 4 Department of Ecology and Sustainable Environmental Management, Lviv Polytechnic National University, UK 79000 Lviv, Ukraine; vira.v.sabadash@lpnu.ua

**Keywords:** polymer inclusion membrane, methylene blue dye, organic dye, calixpyrroles

## Abstract

The effective purification of aqueous solutions of methylene blue dye was tested using polymer inclusion membranes (PIMs) that contained cellulose triacetate (CTA) as a polymer base, o-nitrophenyl octyl ether (*o*-NPOE) as a plasticizer, and *meso*-tetra methyl tetrakis-[methyl-2-(4-acetlphenoxy)] calix[4]pyrrole (KP) as a carrier. Scanning electron microscopy (SEM), thermogravimetric analysis (TGA), and attenuated total reflection Fourier transform infrared (ATR-FTIR) spectroscopy were used to define the microstructure and surface of PIMs. Experimental results showed that, with an increased concentration of methylene blue in an aqueous solution, the removal percentage also increased. Further observation showed that the flux increased with the rise in the source phase pH values from 3 to 10. The carrier and plasticizer content in the membrane significantly influenced the membrane’s transport properties. The optimal composition of the membrane in percent by weight for KP was 74% plasticizer; 18% support, and 8% carrier. The maximum MB removal (93.10%) was achieved at 0.10 M HCl solution as the receiving phase. It was shown that the membrane with optimal composition showed good reusability and enabled the easy and spontaneous separation of methylene blue from aqueous solutions.

## 1. Introduction

One of the most prevalent contaminants in the water environment, methylene blue (MB), is categorized as an organic dye (OD) and discharged as an effluent following a variety of industrial processes [1]. The substrates are dyed to give them a permanent color that will not fade in the presence of water, light, oxidation chemicals, perspiration, or microbiological attack [2]. Because of these qualities, various dyes have numerous applications in the textile, food, rubber, printing, cosmetics, medicine, plastic, concrete, and paper industries [3,4,5]. These industries generate a tremendous amount of wastewater containing carcinogenic and toxic dyes. The effluents pollute water resources, which become unfit for human consumption [6]. Of all industrial branches, the highest quantities of dyes are consumed by the textile industry, with textile dyes being compounds of high complexity and comprising a diverse range of structural groups [7]. They include multiple structural varieties, for example, acidic, reactive, basic, substances that show dispersibility, azo, diazo, anthraquinone-based, and metal-complex dyes. Basic dyes are characterized by solubility in water; they carry a positive charge when in a solution and are subject to electrostatic attraction to negatively charged substrates. Cationic dyes are able to hold either a positive charge that is localized in the NH_4_^+^ group or the charge can be dispersed over the dye cation’s skeletal structure [8].

One of the most widely used substances in the dye industry is methylene blue, a cationic dye with the molecular formula C_16_H_18_N_3_SCl, which is frequently used to color paper, silk, wool, and cotton [9,10,11].

Due to its potential toxicity, the removal of MB from wastewater is currently considered a matter of necessity. Many analytical approaches, such as adsorption processes, precipitation, membrane techniques, *photo*-oxidation and biodegradation, and electrolytic treatments, are applied in the treatment of effluent from textile dyeing [12]. The processes of adsorption and coagulation result in the creation of large amounts of sludge, while heavy metal ions and inorganic salts are highly resistant to being removed through the processes of oxidation and biodegradation. Large quantities of solvents are consumed in the process of removing organic pollutants through the extraction-based separation method. Liquid membrane technology, including polymer inclusion membranes (PIMs), can serve as an interesting alternative to conventional separation methods and could potentially help overcome this drawback [13]. The use of this technology is largely determined by the effectiveness and stability of a polymer membrane material, which depends on the physicochemical properties of the polymer and the ion carrier incorporated in it. Due to their high efficiency and selectivity, modified polymer materials, i.e., those containing immobilized macrocyclic compounds, could constitute an attractive approach for toxic dye removal from wastewater.

PIMs are one of the new types of liquid membranes that, due to their structure and high resistance against elution of an ion carrier, are now more often used in a variety of industries for removing dyes and metal ions from aqueous solutions and upgrading and purifying a variety of elements and chemicals [14,15,16,17,18,19]. The technology is utilized in a wide variety of applications, including pharmaceuticals, agriculture, industrial chemicals, petrochemicals, the food industry, base metal purification, and precious metal refinement. As a result of rapid industrial development and the necessity of utilizing (separating) dyestuffs from aqueous solutions, the study of transport and separation of dyes by PIMs has strongly intensified [8,20,21]. The ability of macrocyclic compounds to selectively complexate organic molecules, their high chemical and thermal stability, and their ease of synthesis from readily available materials have all contributed to the growing body of research on these compounds as possible organic molecule receptors, particularly in recent years.

The design and synthesis of new-generation carriers, particularly calixpyrroles, to increase their practical applications have recently received a lot of attention. Calixpyrroles are macrocycles consisting of pyrrole rings linked by the tetrahedral configuration of carbon atoms through the pyrrolic 2- and -5 positions [22]. A common mechanism of cation bonding in calixpyrroles with ester groups is the cooperative engagement of the cation with both the ionic (ester) group and its cavity. The chemicals indicated above can be used as ion carriers because of this property, which is caused by a change in pH that eliminates the ionic connection and liberates the cation. The co-transport mechanism, which is the process by which changes in pH affect the ion pair released when a ring opens, and the transfer mechanism do not precisely correlate [16,22]. Calixpyrroles are good potential complexing agents because of their highly specific ligand properties and potential applications as hosts and sensing agents for various analytes. Additionally, it is possible to easily modify the scaffold of calixpyrroles using a variety of new compounds to enhance their selectivity and specificity for given cations, anions, and neutral molecules.

PIM containing cellulose triacetate, *meso*-tetra methyl tetrakis-[methyl-2-(4-acetylphenoxy)] calix[4]pyrrole, and *o*-NPOE, used as a matrix, a carrier, and a plasticizer, respectively, was developed and characterized in this study. The obtained membrane can be applied to effectively eliminate MB from an aqueous solution. In order to achieve this, the impact of several parameters, such as the receiving phase’s acidity and the source phase’s pH or MB concentration, on the transport of MB over the PIM was investigated. Investigations were also conducted on the impact of carrier concentration on the transport processes and membrane characterization using SEM, ATR-FTIR, and DSC-TG.

## 2. Materials and Methods

### 2.1. Reagents

Hydrochloric acid (HCl) and sodium hydroxide (NaOH), two examples of analytical-grade chemical reagents, were purchased from POCh in Gliwice, Poland. Analytical-grade organic reagents, i.e., methyl-2-(4-acetylphenoxy)acetate, cellulose triacetate (CTA, molecular weight Mw = 72.000–74.000), *o*-nitrophenyl octyl ether (*o*-NPOE), and dichloromethane (CH_2_Cl_2_) (all from Merck Life Science Sp.z.o.o., Poznan, Poland), were used without further purification. Methylene blue (MB) dye was purchased from Chempur (Krupski Mlyn, Poland). Deionized water (conductivity, 0.10 μS/cm) and analytical-reagent-grade chemicals were used in the production of all aqueous solutions.

### 2.2. Synthesis

**Figure 1:**
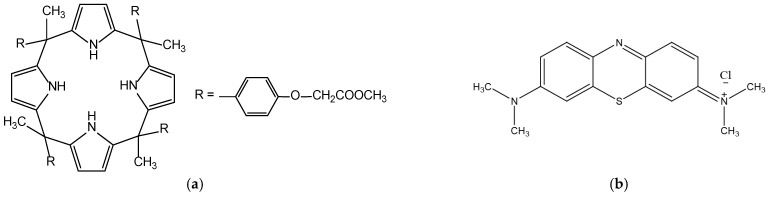
Structures of (**a**) *meso*-tetra methyl tetrakis-[methyl-2-(4-acetlphenoxy)] calix[4]pyrrole (KP), (**b**) methylene blue (MB).

The synthesis of *meso*-tetra methyl tetrakis-[methyl-2-(4-acetylphenoxy)] calix[4]pyrrole (KP) (Figure 1) was carried out using a pyrrole and ketones reaction, with acids acting as catalysts. The condensation products were primarily four-membered calixpyrrole ring isomers. The *meso*-tetra methyl tetrakis-[methyl-2-(4-acetylphenoxy)] calix[4]pyrrole from pyrrole and methyl-2-(4-acetylphenoxy)acetate according to data available in the literature [23] was obtained. Methyl-2-(4-acetylphenoxy)acetate was poured into a round-bottomed flask with pyrrole in dry CH_2_Cl_2_, methyl-2-(4-acetylphenoxy)acetate. The resulting mixture was subjected to cooling to 0 °C and later stirred for 5 min. Through a 10 min process of bubbling using Ar, the mixture resulting from the reaction was degassed. Afterwards, for another 10 min in a nitrogen atmosphere, HCl was added dropwise over the mixture. The solution created through this process was subjected to stirring for 2 h at 0 °C and then stored overnight at room temperature. After removing the solvent, ethyl acetate was used in order to dissolve the crude product. The resulting solid was subjected to multiple washing cycles using water and dried with MgSO_4_. Chromatographic purification (silica gel, chloroform/methanol: 1/1) yielded a white solid (80%). A Bruker Advance 3HD 400 MHz spectrometer working at 400 and 100 MHz for ^1^H and ^13^C, respectively, was used to record the NMR spectra. NMR spectra were recorded in a chloroform-d (CDCl_3_), 99.8% atom %D solution. The NMR spectra are available in the Supplementary Material.

The structure of KP was confirmed by ^1^HNMR (Bruker Advance, 400 MHz, CDCl_3_, 298 K, ppm): 7.53–7.46 (s; 4H, NH); 6.90–6.88 (d, 8H, ArH); 6.69–6.67 (d, 8H, ArH); 5.85–5.66 (d, 8H, PyH); 4.52 (s, 8H, CH_2_); 1.8 (s, 12H, CH_3_); 1.2 (s, 12H, CH_3_) and ^13^C NMR (Bruker Advance, 101 MHz, CDCl_3_, 298 K, ppm): δ 168.95, 156.36, 140.73, 136.85, 136.61, 128.47, 113.96, 106.1, 105.49, 77.34, 77.02, 76.71, 67.95, 65.50, 61.34, 58.41, 44.10, 29.69, 29.61, 18.35, 14.18.

### 2.3. Preparation of PIMs and Stability Test

The preparation of PIMs was carried out following a procedure previously described by Nowik-Zajac [16]. The following solutions were used in order to prepare PIMs (in CH_2_Cl_2_, an organic solvent): cellulose triacetate (CTA) serving a base polymer role, *o*-nitrophenyl octyl ether used as a plasticizer, and *meso*-tetra methyl tetrakis-[methyl-2-(4-acetylphenoxy)] calix[4]pyrrole (KP) (Figure 1) performing the function of carrier. The volumes of CTA and plasticizer solutions were fixed at 2.0 and 1.0 mL, respectively. The volumes of carriers were in the range of 0.10 to 1.0 mL. After mixing the defined CTA solution, plasticizer, and carrier volumes, the resulting mixture was moved to a 5.0 cm glass ring bonded to a plate glass using 5-dichloromethane glue. The glass ring was allowed to sit at room temperature for the entire night in order to allow the CH_2_Cl_2_ to evaporate. By wetting it in cold water, the membrane obtained as a result of this process was detached from the glass plate. The effective area of the membrane was 4.9 cm^2^. The thickness of the membrane was measured with a digital micrometer (PosiTector 6000 Advance, DeFelsko Corporation, New York, NY, USA) over four readings, with an accuracy of 1.0 μm standard deviation. The CTA membrane’s average thickness was 24 μm.

The stability of PIMs was examined with the loss of mass in mind, which is associated with plasticizer and/or carrier leaching. For that purpose, PIMs were subjected to immersion in 100 mL of ultrapure water and 24 h of shaking. Prior to and after this procedure, the membranes were weighed, and the weighing data served as a basis for calculating the mass loss. Examination of membrane masses was also performed prior to and after the MB transport.

### 2.4. Transport Studies

The transport tests were carried out in a permeation cell with a two-compartment setup, as described in our previous study [16], where the membrane film was firmly clamped between the receiving and source phases. MB (50 mL) served as the source phase, whereas hydrochloric acid (50 mL) performed the function of the aqueous receiving phase. The experimental procedures were performed at room temperature (23 to 25 °C), and synchronous stirrers were used to agitate both the source and receiving aqueous phases at 600 rpm. Aqueous phase samples were periodically removed through a sampling port using a syringe and were subjected to analysis in order to ascertain the concentration of MB. The acidity and basicity of both aqueous phases were controlled by a pH meter (multifunctional pH meter, CX-731 Elmetron, with a combined pH electrode, ERH-136, Hydromet, Zabrze, Poland).

The parameters describing transport, such as the rate constant *k*, permeability *P*, and initial flux *J_i_*, were calculated from the following relationships [24]:

(1) $$\ln\left(\frac{c}{c_{i}}\right)=-kt$$

(2) $$P\;=-(\frac{V}{A})k$$

(3) $$\;\;\;\;\;\;J_{i}=P\;c_{i}$$

where *c* is the MB concentration (mol/dm^3^) in the source phase at a given time, $c_{i}$ is the initial MB concentration in the source phase (mol/dm^3^), *k* is the rate constant (s^−1^), *t* is the transport time (s), *V* (cm^3^) is the volume of the aqueous source phase, and *A* (cm^2^) is the area of the membrane.

To describe the efficiency of MB removal from the source phase, the recovery factor (*RF*) was calculated as follows:

(4) $$RF=\left(\frac{c_{i}-c}{c_{i}}\right)100\%$$

Within the scope of transport experiments, the quantification of MB was performed using a UV/Vis spectrophotometer (Shimadzu 2401, Shimadzu, Kyoto, Japan). On the basis of prior studies concerning methylene blue [8], there was no effect of the aqueous solution’s pH on the MB absorption of the characteristic band in UV/Vis spectroscopy by UV/Vis at λ = 663 nm.

### 2.5. Characteristics of Developed Polymer Inclusion Membranes (PIMs)

The thickness of the PIM samples was measured using a digital micrometer (PosiTector 6000 Advance, DeFelsko Corporation, New York, NY, USA) with an accuracy of 0.1 µm. For every case, the membrane’s thickness was measured ten times, and the average measurement was provided with a standard variation of less than 1%. The thickness of membranes before and after transport was found to be the same. The average PIM thickness was 25 µm. Experimental reproducibility was high, with a standard deviation below 1% of the measured values.

#### 2.5.1. Morphology

The KP network’s membrane morphology was examined using a 5 KV scanning electron microscope (SEM) (Quanta 3D FEG, FEI Company, Hillsboro, OR, USA) (Hitachi S4500, Hitachi, Tokyo, Japan). The membrane samples were frozen in liquid nitrogen (70 K) and subjected to rapid fracturing. Subsequently, the samples were adhered to metal stubs featuring shattered edges using conductive glue, and a gold coating was applied via the sputtering technique. Then, SEM inspection of the samples was performed at a magnification of approximately 50 μm.

#### 2.5.2. Analysis of Thermal Properties: Differential Scanning Calorimetry (DSC), Thermogravimetric Analysis (TGA) of PIMs

Thermal analysis of the membranes was conducted using differential scanning calorimetry (DSC) and thermogravimetric analysis (TGA), incorporating a simultaneous analyzer for DSC–TGA research (STA 409C, Netzsch, Selb, Germany) to determine their heat resistance. The analysis was carried out under a nitrogen flow of 100 mL/min and at a heating rate of 10 °C/min. The TGA experiment was performed in a temperature range of 30–450 °C, and the samples were placed in sealed, non-hermetic aluminum pans. The DSC experiments were conducted in a temperature range of 30–450 °C, and the samples were placed in sealed aluminum pans. The TGA analysis was described as a mass percentage change during heating and as differential thermogravimetry (DTG), which made it possible to accurately identify the exhibited processes. The DSC analysis was described to indicate phase transition behavior dependent on heating.

#### 2.5.3. Analysis of Structure: Attenuated Total Reflection Fourier Transform Infrared (ATR-FTIR) Spectroscopy of PIMs

The ATR-FTIR spectra were recorded using a Bruker Alpha-PFT-IR device with a diamond attenuated total reflectance (ATR) accessory and were recorded within a wavenumber range of 500–4000 cm^−1^.

## 3. Results and Discussion

### 3.1. Kinetics and Repeatability of the Methylene Blue (MB) Dye Transport across Polymer Inclusion Membranes (PIMs)

**Figure 2:**
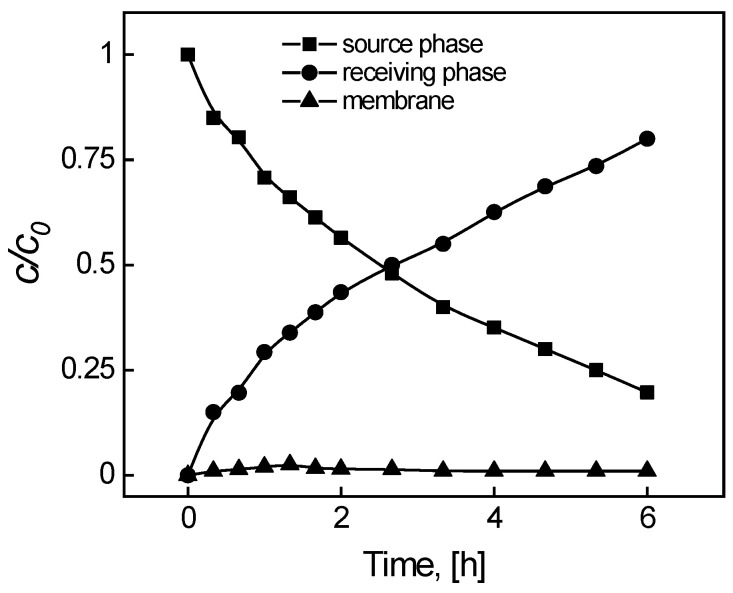
The profiles of MB concentrations in the source, membrane, and receiving phases during transport across PIM containing KP. Source phase: 100.0 μM MB, pH_source phase_ = 10.0; membrane: 2.0 mL *o*-NPPE/1.0 g CTA; 0.050 M KP, receiving phase: 0.10 M HCl.

Measurements of the dye concentration changes in the source and receiving phases as a function of the transport time through the membrane were made in order to formulate a concentration change profile over time and determine the MB separation process kinetics and repeatability of the PIM containing KP. The primary transport kinetic model for metal ions in connection to supported liquid membranes (SLM) [24] is reflected in the exponential *c*/*c_i_* relationship curves in Figure 2 as a function of time, as suggested by Danesi et al. [24].

The rates of transport at the source phase and membrane boundary, as well as the boundary of the membrane and receiving phase, are comparable, i.e., there is an equivalent rate of complexation and decomplexation reactions.

**Figure 3:**
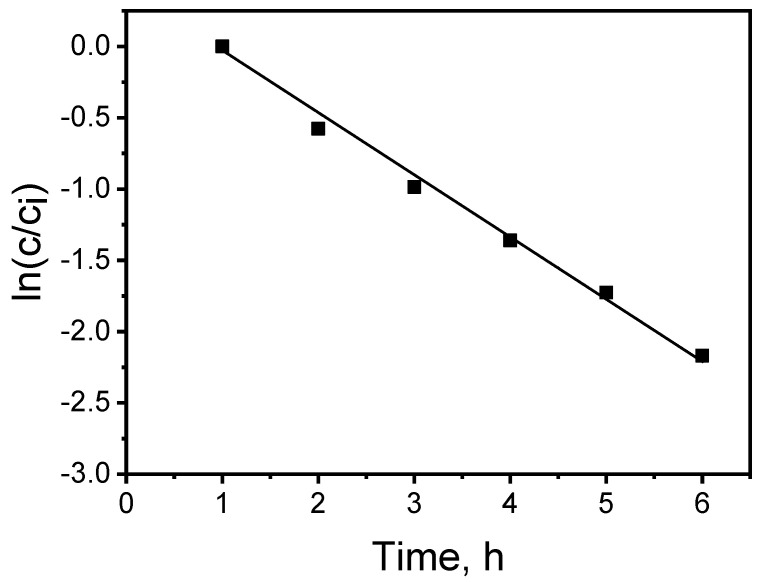
The relationship ln(*c*/*c_i_*) vs. transport time of MB across the PIM with KP. Source phase: 100.0 μM MB, pH_source phase_ = 10.0; membrane: 2.0 mL *o*-NPPE/1.0 g CTA; 0.050 M KP, receiving phase: 0.10 M HCl.

The estimation of the average transport process constants (*k*) was performed on the basis of the linear relationship slopes ln(*c*/*c_i_*) = *f*(*t*) obtained for the PIM used (Figure 3) and served as a basis for calculating the MB initial flux values from the source phase (*J_i_*).

The determination coefficient (*r*^2^) of the linear relationship ln(*c/c_i_*) as a function of time for the KP carrier was 0.9962, which pointed to a correlation with a high level of significance. The relative error burden of the obtained results did not exceed 1% and was simultaneously obtained with a high determination coefficient value.

### 3.2. The Effect of Carrier Concentration

The role and composition of the carrier are important in the context of the transportation of the MB dye through the PIM. Hence, to improve transport efficiency, it is necessary to optimize the relative KP content in the liquid membrane [16].

The effect of the carrier quantity in the PIM on MB dye permeation was examined. Membranes with a fixed content of CTA (25 mg) and plasticizer (2 mL *o*-NPPE/1.0 g CTA) were prepared, while the concentration of carrier in the membrane was changed in the range of 0.010 to 0.10 M (based on plasticizer volume). Each experiment was conducted for 6 h. The fact that MB could not be carried by membranes in the absence of a carrier suggests that MB transport through the PIM depends critically on the carrier concentration.

**Figure 4:**
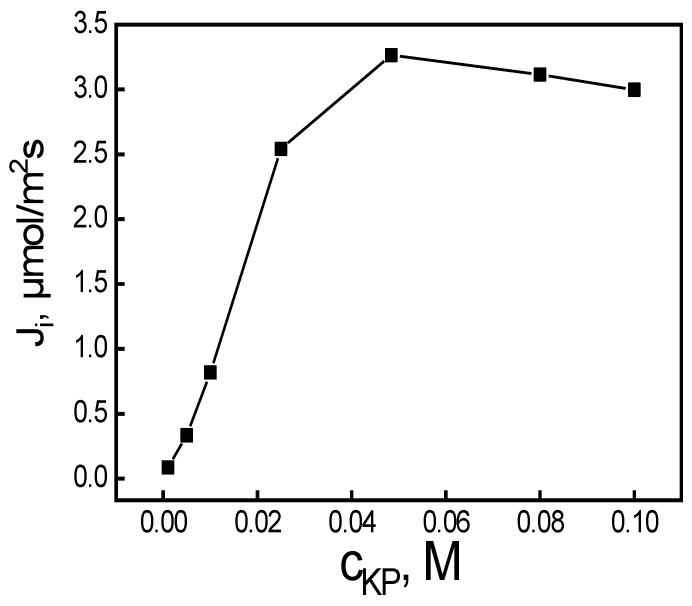
The MB transport fluxes vs. carrier concentration in the PIM. Source phase: 100.0 μM MB, pH_source phase_ = 10.0; membrane: 25 mg CTA; 2.0 mL *o*-NPPE/1.0 g CTA; receiving phase: 0.10 M HCl.

The lower KP content in the PIM was insufficient to allow MB transport through the membrane. The transport efficiency increased with the KP content of the membrane to 0.050 M (Figure 4). At a KP carrier concentration of 0.050 M in the membrane (based on the plasticizer volume), the PIM became saturated with the carrier. At that carrier concentration, the calculated transport rate reached 3.42 μmol/m^2^s, the maximum value. Further KP content increase did not cause any transport efficiency enhancement; it rather decreased by about 12% beyond 0.050 M, which can be a result of steric hindrance due to excess KP [25]. The rise in viscosity of the organic membrane phase, which results in membrane resistance, thus restricting complex diffusion through the membrane, is the explanation provided for this phenomenon in previous research [26,27,28]. Or it may be that there is a transport mechanism change from diffusion to jumping, which is induced by the process of carrier crystallization within the membrane [27]. The membrane’s low wettability reduces its permeability, which consequently leads to a low transport rate. Hence, we selected 0.050 M KP for the PIM synthesis for further studies. The optimal composition of the membrane in percent by weight for KP was 74% plasticizer, 18% support, and 8% carrier.

### 3.3. Modification of Source Phase Composition

**Figure 5:**
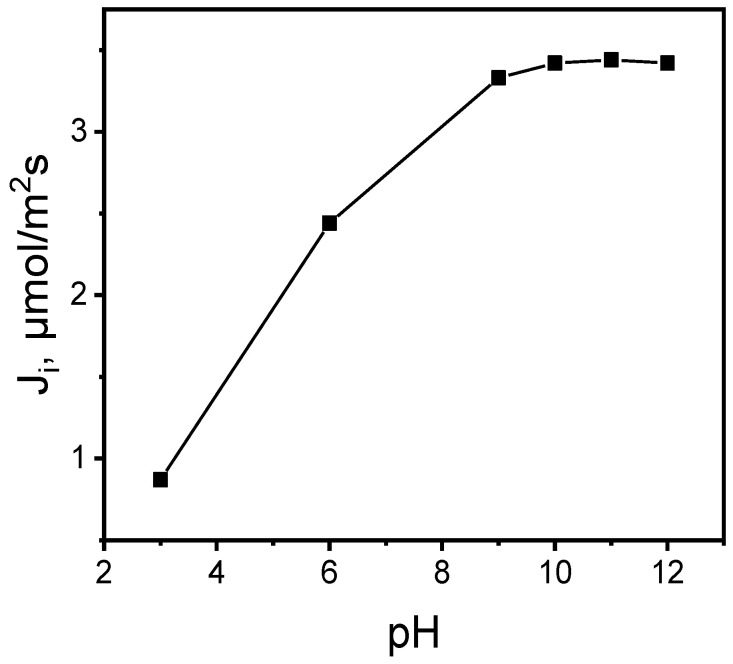
The relationship between the initial MB ion flux transport across the PIM and the pH of the source phase. Source phase: 100.0 μM MB; membrane: 2.0 mL *o*-NPPE/1.0 g CTA; 0.050 M KP, receiving phase: 0.10 M HCl.

The effect of the pH source phase within the range of 3.0–12.0 on the MB dye transport through the PIM was examined. Figure 5 shows the initial fluxes of the MB dye transport as a function of pH from the source phase (100.0 μM MB dyes) via the PIM to the receiving phase (0.10 M HCl).

As was shown, as the source phase pH rose from 3.0 to 10.0, the values of the initial flux transport increased. The maximal MB ion transport initial flux value for pH = 10.0 was 3.42 μmol/m^2^s from the aqueous source phase containing 100.0 μM of MB through the PIM; however, the transport flux was stable within a pH range of 10.0–12.0. The PIM containing an ester derivative called calix[4]pyrrole significantly affected the MB transport rate by virtue of the aqueous phase basicity. The findings were that raising the pH (up to 10) increased the effectiveness of MB transport (Table 1); an increase in the aqueous source phase solution’s pH correspondingly resulted in raising the initial MB flux when the receiving phase HCl concentration equaled 0.10 M. The ester group’s impact causes the initial flow to increase at a higher pH, which improves the synthesis of extractable target complex species in the organic membrane phase, which occurs as a counterion. The esters’ essential nature arises from the ester group’s C=O bond polarization. Indeed, MB absorption from the bulk solution rises with pH in the base region. The repulsion between extracting sites and MB may be the reason for the lower absorption capacity in the acidic zone, whereas the hydrophobic MB conversion into hydrophilic ester–methylene blue may account for the higher absorption capacity observed at a base pH. Therefore, the KP carrier interacts with the ester–methylene blue intermediate complex. However, because there are more polar species groups present, a pH of 10 is better for extraction. While the counterions are transferred in the opposite direction, the MB–KP carrier complex is moved from the source to the receiving phase via the PIM. As the increase in the pH of the aqueous source phase up to 10 causes a rise in MB transport, the pH gradient becomes a driving force for MB transport across the PIM. In their studies using calix[4]resorcinarene as a carrier, Benosmane et al. observed that the flux value from the aqueous source phase increased as the pH value increased from 3 to 11. It was also found that the carrier content in the membrane has a significant impact on the properties of PIM [15].

**Figure 6:**
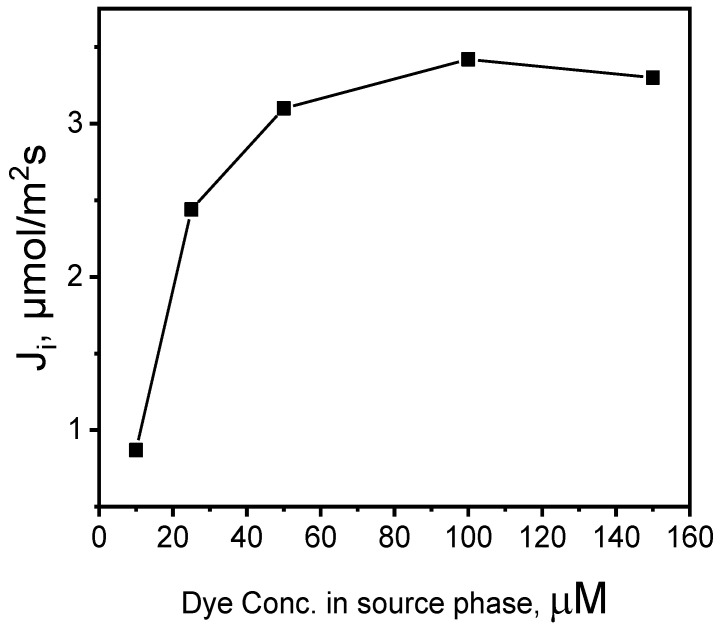
The effect of MB concentrations in the source aqueous phase. Source phase: various concentrations of MB, pH_source phase_ = 10.0; membrane: 2.0 mL *o*-NPPE/1.0 g CTA; 0.050 M KP, receiving phase: 0.10 M HCl.

Five MB solutions at pH 10.0 containing 10.0, 25.0, 50.0, 100.0, and 150.0 μM of MB were prepared, which were used as the source phases for the purpose of determining the PIM process kinetics due to the MB concentration in the source phase. The 0.10 M HCl was used as the receiving phase. The impact of the initial MB dye concentrations on the transport efficiency through the PIM in the source phase is presented in Figure 6.

The initial flux increased with the rise in MB dye concentration up to 100.0 μM; its further growth, however, did not improve the transport efficiency. The increased MB concentration results in membrane saturation, which in turn decreases the effective area available for transportation and hinders MB cation mobility within the source phase [29]. Therefore, a 100.0 μM solution of MB was selected to optimize other parameters of the experiment.

### 3.4. Modification of the Receiving Phase Composition

The impact of varying hydrochloric acid concentrations (0.1–1.0 M HCl) in the receiving phase was examined in relation to the flow of MB via the PIM (Table 2).

The MB transport efficiency across the PIM is increased by the receiving phase’s decrease in acid concentration. The maximum MB removal (93.10%) was achieved at 0.10 M HCl solution as the receiving phase. An explanation of these results lies in the high acid concentration that causes a reduction in the dissociation of the carrier–dye complex formed within the liquid membrane.

### 3.5. Membrane Reusability and Proposed Transport Mechanism

The biggest benefit of PIMs, besides their other qualities, is their reusability, which guarantees their industrial application [19]. In the repeated MB transport (six cycles), the same PIM membrane was used, while both aqueous phases were replaced for each cycle.

**Figure 7:**
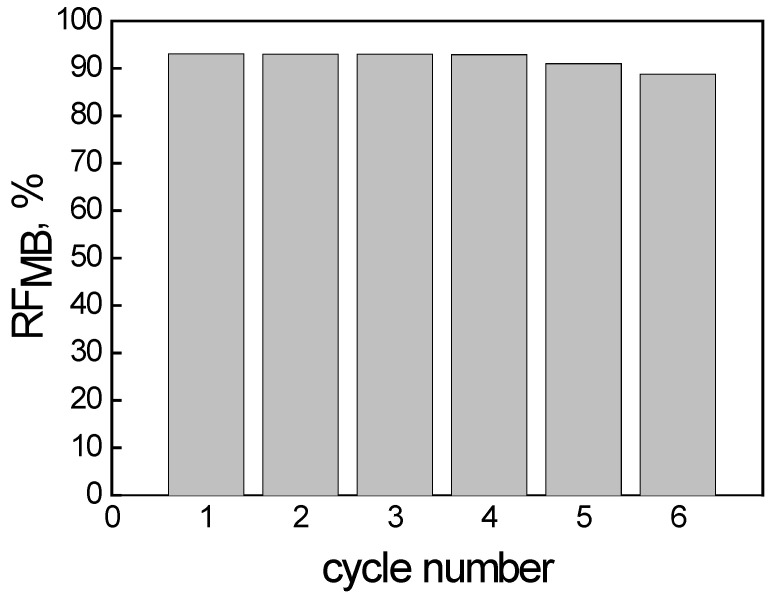
The recovery factor (RF) of MB vs. the cycle number. Source phase: 100.0 μM MB, membrane: 18 wt.% of CTA, 8 wt.% of KP, 74 wt.% of *o*-NPOE; receiving phase: 0.10 M HCl.

The RF values (Figure 7) exceeded 93% in the first four transport cycles (each cycle lasting 6 h), then dropped inconsiderably (to approx. 90%). The obtained recovery factors (RF) indicate that the efficiency of MB removal by the PIM is repeatable. The PIM with an optimal composition seems to be effective when used repeatedly in the process of removing MB.

Because MB is a cationic dye, as is exhibited by its structural pattern (Figure 1b), it is deprotonated in the source phase into the non-ionic form owing to the reaction of MB^+^ with OH^−^ ions. The deprotonated MB easily diffuses into the membrane and, more precisely, is complexed with the KP carrier. The complexation is based on the inclusion complex between the KP as a host and the MB as a guest molecule. The guest molecule is inserted into the host cavity, in this case, pyrrole units and an alkyl chain with ester-bound cover dye molecules, presumably. Hydrophobic interactions, hydrogen bonding, and slightly π-electron interactions could behave as binding forces. Diffusion through the membrane may result in the release of MB into the receiving phase, but only in an acidic environment, according to the experimental data. It is connected to the protonation of the MB, which results in decomplexation with the KP. Then, the MB molecule becomes positively ionized and cannot be preserved due to the decreasing strength of hydrophobic interactions [20]. The proposed mechanism of MB separation through the PIM containing the KP is dependent on the pH gradient as the driving force.

### 3.6. Physical–Chemical Characterization of the Developed PIMs

#### 3.6.1. Morphology of the Developed PIMs

**Figure 8:**
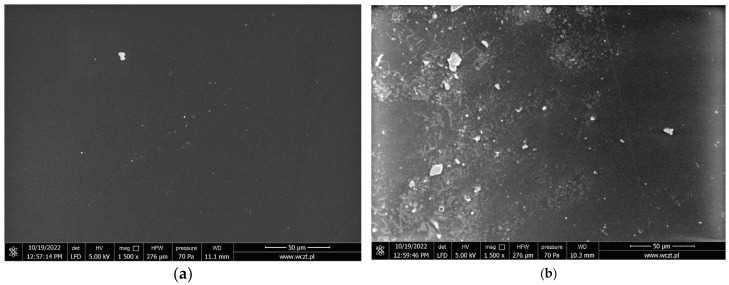
SEM images of PIMs containing KP (magnification 50 µm) (**a**) before transport MB, (**b**) after transport MB.

The SEM images of the PIM based on the mixture of 0.01 g KP, 2.0 mL CTA, and 1.0 mL *o*-NPOE before and after the transport of MB are presented in Figure 8.

Because of the MB accumulation in the membrane phase as a result of the PIM’s plasticization, a comparison of surface microstructure shows the variation in membrane matrix materials in terms of amount and sediment distribution [30]. Image (a) corresponds to the surface of the membrane before transporting MB and clearly shows that the surface is homogeneous and smooth without apparent porosity. In contrast, the membrane after transport MB exhibits a clear porous membrane structure covered with small particles found on partially smooth surfaces. Furthermore, the images illustrate a perceivable film surface roughness. It is possible for the crystallization of carriers to occur in the membrane.

#### 3.6.2. Thermal Properties Analysis Results

**Figure 9:**
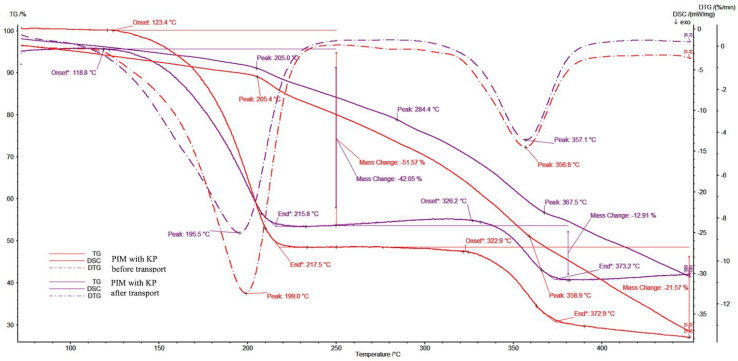
Thermogram analysis of the developed PIMs.

The TGA and DSC analyses were conducted simultaneously with the addition of DTG in order to accurately explain the thermal PIM degradation processes. The resulting thermograms (Figure 9) exhibited long mass losses up to 118 °C, related to the slow dehydration of approximately 13% of the mass. The most significant mass change began at 196 °C, with the loss of half of the mass, according to the DTG. The curve change is related to a depolymerization process resulting in KP thermos degradation and ester linkage cleavage [31]. Thermos degradation of KP occurred at 220 °C. Depolymerization ended at about 320 °C, where the DTG curve indicates the appearance of another important process lasting up to 360 °C, which is responsible for the thermodegradation of phenyl units.

The thermal decomposition of the examined PIM is an irreversible process, observed in the first and second stages as being influenced by externally supplied heat and, in further stages, by the heat released during combustion. According to the information available in the literature, the primary distribution of unplasticized CTA matrix takes place at temperatures of 292–320 °C, which corresponds to the breaking of the polymer chains [32,33], while CTA membranes plasticized with *o*-NPPE exhibit thermal stability only to approximately 200 °C [34]. A conclusion can, therefore, be drawn that introducing KP increased the thermal stability of our PIM.

#### 3.6.3. ATR-FTIR

**Figure 10:**
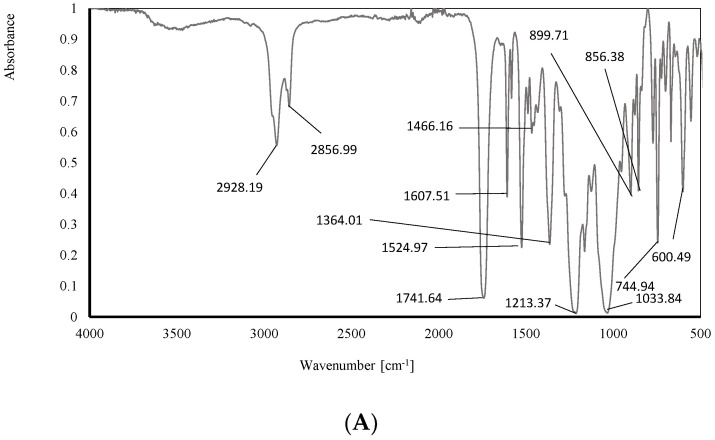

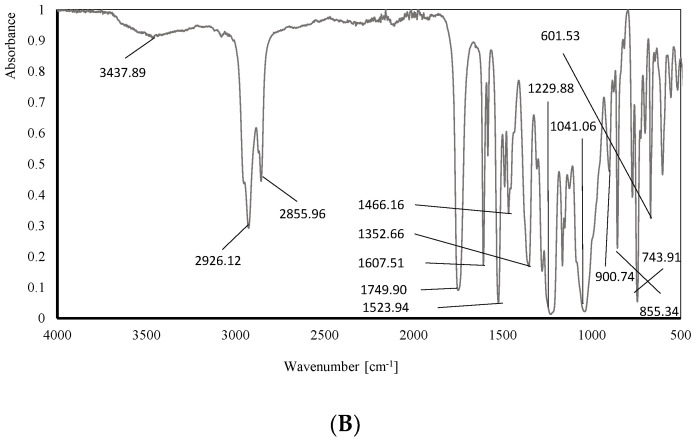
The infrared spectrum of membrane (**A**) before and (**B**) after the transport process.

The following figures show the infrared spectra of the investigated membranes with optimal composition before (Figure 10A) and after (Figure 10B) transport processes.

The interpretation of the above infrared spectra was made using the IRPal 2.0 table-driven infrared application. The most intensive bands identified in the ATR-FTIR spectra are summarized in Table 3.

The visibility of all characteristic bands on the above ATR-FTIR spectra confirms the presence of individual components in the membranes: a polymer—CTA, a plasticizer—*o*-NPOE, and the carriers—meso-tetra methyl tetrakis-[methyl-2-(4-acetlphenoxy)] calix[4]pyrrole (KP). The ATR-FTIR spectra of both membranes are similar, and that evidences stability during/after the transport process. Carrier and any other components did not wash out of the structures of the membrane.

## 4. Conclusions

It was possible to successfully utilize the developed CTA-based polymer inclusion membrane (PIM) in the role of base polymer, as well as *meso*-tetra methyl tetrakis-[methyl-2-(4-acetlphenoxy)] calix[4]pyrrole (KP) functioning as a complexing agent in the recovery of methylene blue (MB) from the aqueous solution. According to experimental data, the proportion of MB removed from wastewater streams increased when the MB concentration was increased from 10 to 100 µM. It was also noted that when pH values rose from 3 to 10, the flux increased. The carrier’s content in the membrane significantly influenced the characteristics of PIMs. The highest flux value of 3.42 μmol/m^2^s was obtained with 0.05 M KP as the carrier in the PIM at pH = 10.0 in the source and 0.10 M HCl in the receiving phases when the initial MB concentration was 100 μM. The PIM we have developed exhibited good reusability and high MB removal efficiency (93.10%) from aqueous solutions.

## Figures and Tables

**Table 1:** The effect of the source phase pH on the removal of MB when the receiving phase is 0.1 M HCl.

pH of Source Phase	% Removal of MB
3	5.48
6	18.43
9	74.57
10	93.10
11	92.12
12	91.89

**Table 2:** The effect of acid concentration in the receiving phase.

Concentration of HCl in the Receiving Phase	% Removal of MB
0.1 M	93.10
0.2 M	89.75
0.3 M	88.48
0.5 M	70.58
1.0 M	65.54

**Table 3:** Interpretation of the obtained ATR-FTIR.

Bond	Membrane before the Transport Process (A)	Membrane after the Transport Process (B)	Typical Absorption Range [cm^−1^] *
	Wavenumber [cm^−1^]		
R-X alkyl halides	600.50	601.53	500–680
C-H aromatics	744.94	743.91	705–745
C-H aromatics	856.37	855.34	862
N-H amine groups	899.71	900.74	665–910
RCO-OH carboxylic groups RCOOR’ C-O	1033.84	1041.06	1000–1320
Ar-O-R ether groups	1213.37	1229.88	1220–1260
C-H alkanes	1364.01	1352.66	1360, second 723
RCH_2_CH_3_ alkanes CH_2_. CH_3_	1466.16	1466.16	1460
N-O nitro groups	1524.97	1523.94	1520, second 1350
C=C alkenes	1607.51	1607.51	1611
RCOOR’ esters groups	1741.64	1749.90	1735
-CH_2_- alkanes C=C CO-OH carboxylic groups dimer OH	2856.99	2855.96	2850 2800–3400
-CH_2_- alkanes	2928.19	2926.12	2925
RCONHR’	3437.89	3437.89	3440

* Data source: IRPal 2.0 table-driven infrared application.

## Data Availability

The original contributions presented in the study are included in the article, further inquiries can be directed to the corresponding author.

## Supplementary Materials

The following supporting information can be downloaded at: https://www.mdpi.com/article/10.3390/membranes14040092/s1, Figure S1: The NMR spectra.
