# Supplementary material for: Removal of Methylene Blue Dye from Aqueous Solutions Using Polymer Inclusion Membrane Containing Calix[4]pyrrole
Source: Membranes (Basel). 2024 Apr 17;14(4):92. doi: 10.3390/membranes14040092 (PMC11051798; doi:10.3390/membranes14040092)

The NMR spectra

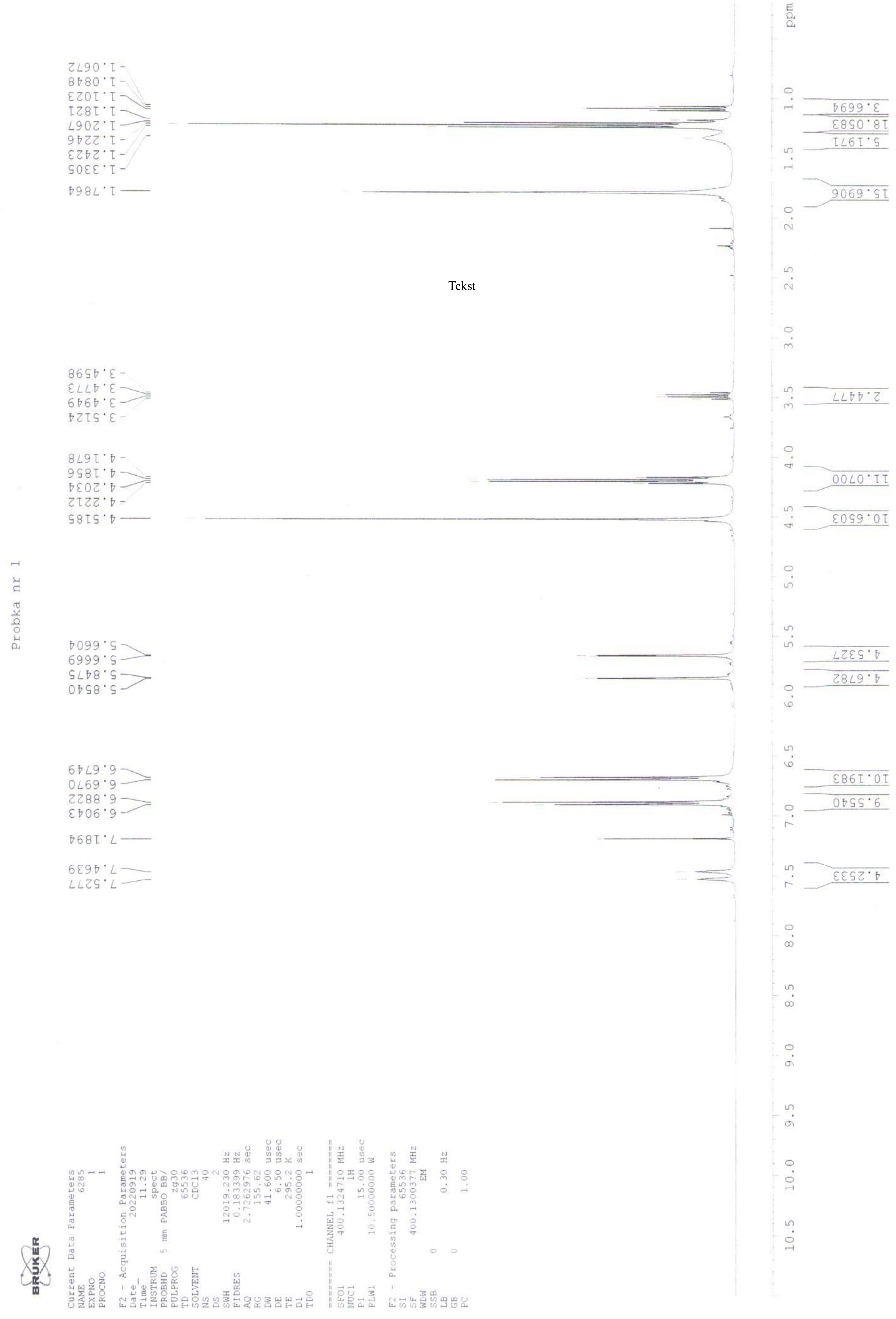

Tekst

Probka nr 1

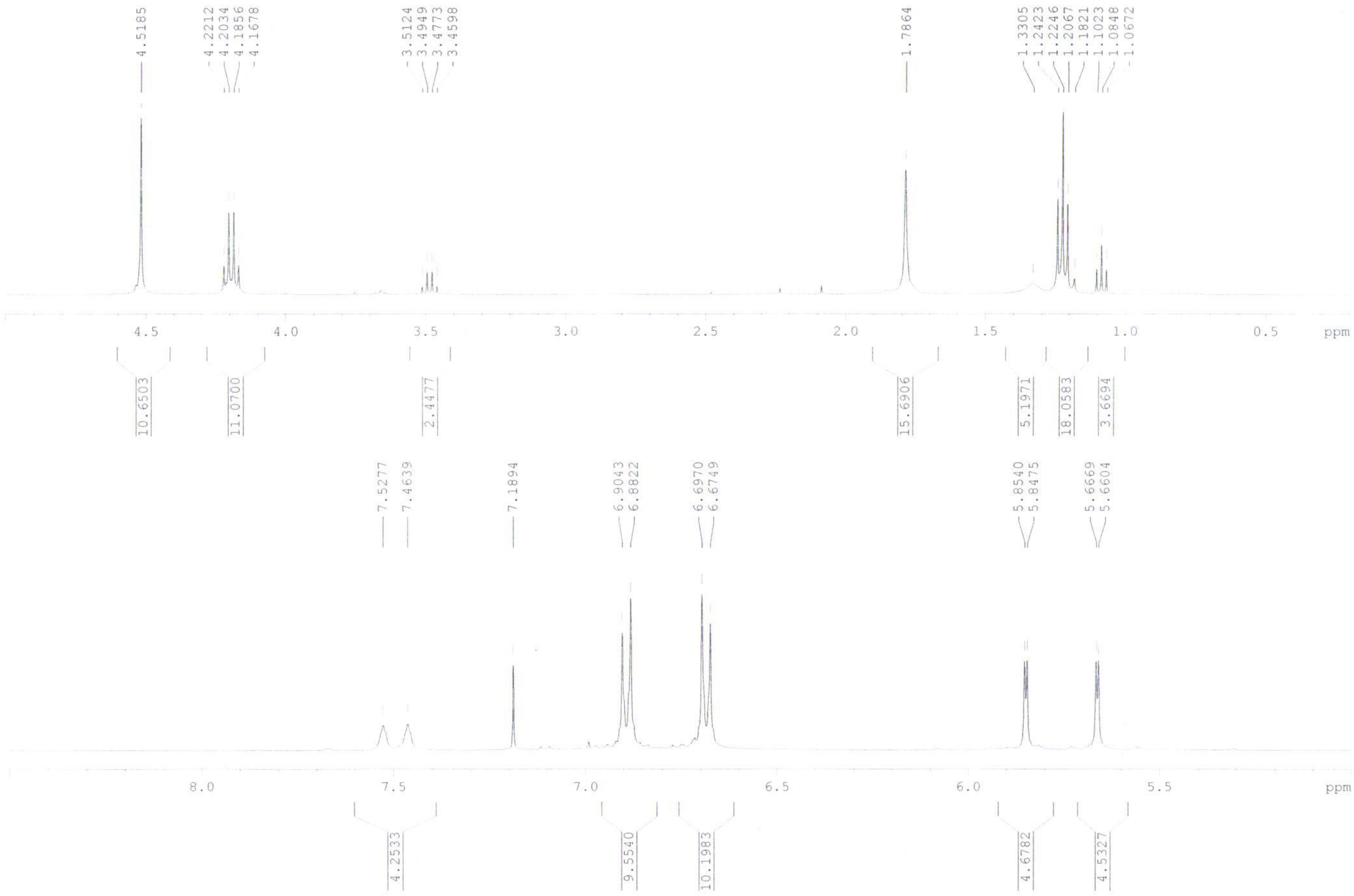
4.5185
4.2212
4.2034
4.1856
4.1678
3.5124
3.4949
3.4773
3.4598
1.7864
1.3305
1.2423
1.2246
1.2067
1.1821
1.1023
1.0848
1.0672
4.5
4.0
3.5
3.0
2.5
2.0
1.5
1.0
0.5
ppm
10.6503
11.0700
2.4477
15.6906
5.1971
18.0583
3.6694
7.5277
7.4639
7.1894
6.9043
6.8822
6.6970
6.6749
5.8540
5.8475
5.6669
5.6604
8.0
7.5
7.0
6.5
6.0
5.5
ppm
4.2533
9.5540
10.1983
4.6782
4.5327

Probka nr 1_13C

Current Data Parameters

NAME	6285
EXPNO	2
PROCNO	1

F2 - Acquisition Parameters

Date_	20220919
Time	14.21
INSTRUM	spect
PROBHD	5 mm PABBO BB/
PULPROG	zgpg30
TD	65536
SOLVENT	CDCl3
NS	25100
DS	4
SWH	29761.904 Hz
FIDRES	0.454131 Hz
AQ	1.1010048 sec
RG	2050
DW	16.800 usec
DE	6.50 usec
TE	296.0 K
D1	2.00000000 sec
D11	0.03000000 sec
TD0	1

======== CHANNEL f1 ========

SFO1	100.6228293 MHz
NUC1	13C
P1	10.00 usec
PLW1	50.00000000 W

======== CHANNEL f2 ========

SFO2	400.1316005 MHz
NUC2	1H
CPDPRG[2	waltz16
PCPD2	90.00 usec
PLW2	10.50000000 W
PLW12	0.29166999 W
PLW13	0.23625000 W

F2 - Processing parameters

SI	32768
SF	100.6127685 MHz
WDW	EM
SSB	0
LB	1.00 Hz
GB	0
PC	1.40

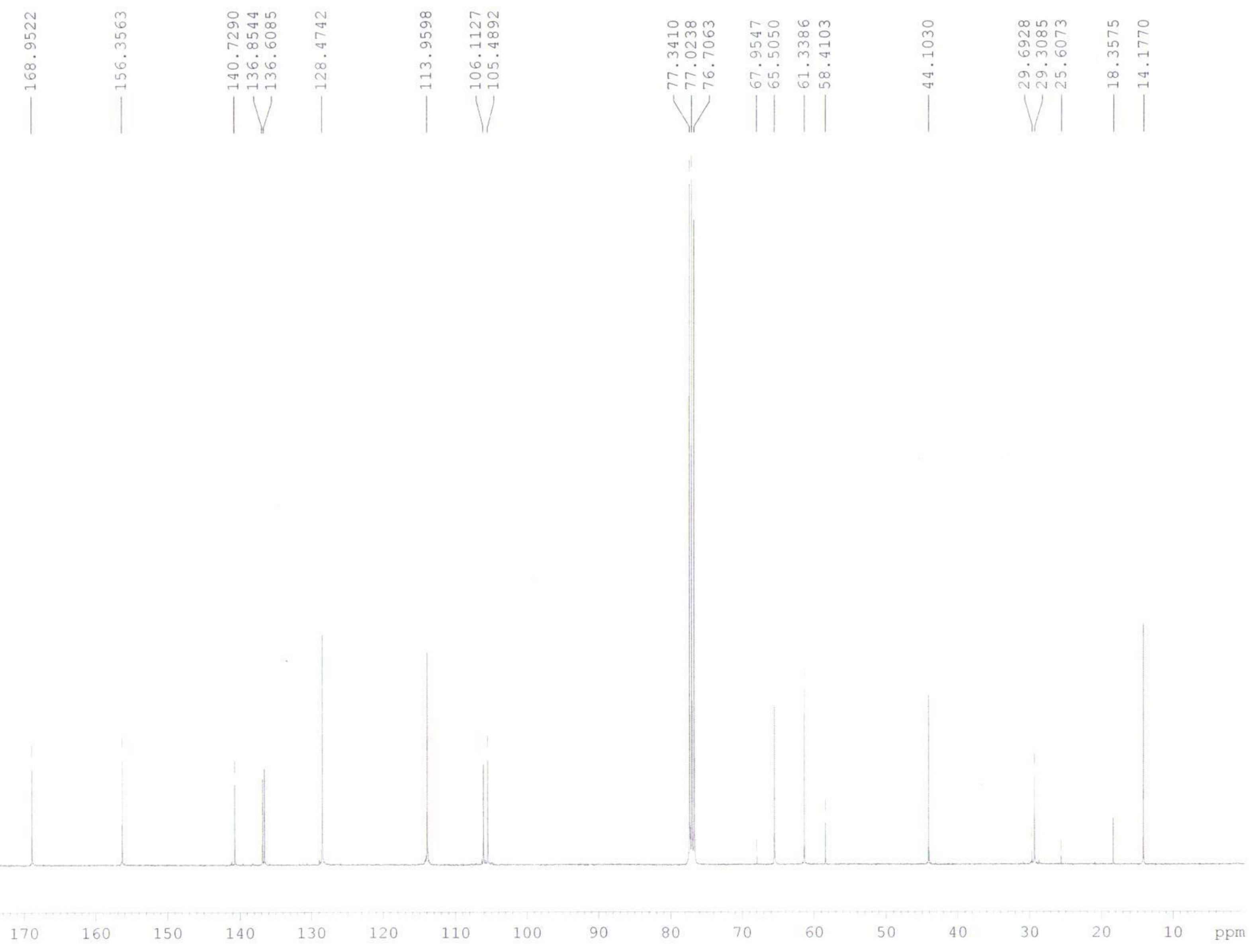

Supplement: Supplementary file 1 [file membranes-14-00092-s001.zip › membranes-2933402-supplementary-Figure S1.pdf]
